# Cytotoxic effects of Gemcitabine-loaded liposomes in human anaplastic thyroid carcinoma cells

**DOI:** 10.1186/1471-2407-4-63

**Published:** 2004-09-13

**Authors:** Marilena Celano, Maria Grazia Calvagno, Stefania Bulotta, Donatella Paolino, Franco Arturi, Domenicoantonio Rotiroti, Sebastiano Filetti, Massimo Fresta, Diego Russo

**Affiliations:** 1 Department of Pharmacobiological Sciences, University of Catanzaro "Magna Græcia", 88100 Catanzaro, Italy; 2 Department of Pharmaceutical Sciences, University of Catania, 95100 Catania, Italy; 3 Department of Clinical Sciences, University of Roma "La Sapienza", 00100 Roma, Italy

## Abstract

**Background:**

Identification of effective systemic antineoplastic drugs against anaplastic thyroid carcinomas has particularly important implications. In fact, the efficacy of the chemotherapeutic agents presently used in these tumours, is strongly limited by their low therapeutic index.

**Methods:**

In this study gemcitabine was entrapped within a pegylated liposomal delivery system to improve the drug antitumoral activity, thus exploiting the possibility to reduce doses to be administered in cancer therapy. The cytotoxic effects of free or liposome-entrapped gemcitabine was evaluated against a human thyroid tumour cell line. ARO cells, derived from a thyroid anaplastic carcinoma, were exposed to different concentrations of the drug. Liposomes formulations were made up of 1,2-dipalmitoyl-sn-glycero-3-phosphocholine/cholesterol/1,2-distearoyl-sn-glycero-3-phosphoethanolamine-MPEG (8:3:1 molar ratio). Cell viability was assessed by both trypan bleu dye exclusion assay and fluorimetric analysis of cell DNA content.

**Results:**

A cytotoxic effect of free gemcitabine was present only after 72 h incubation (ARO cell mortality increased of approximately 4 fold over control at 1 μM, 7 fold at 100 μM). When gemcitabine was encapsulated in liposomes, a significant effect was observed by using lower concentrations of the drug (increased cell mortality of 2.4 fold vs. control at 0.3 μM) and earlier exposure time (24 h).

**Conclusion:**

These findings show that, *in vitro *against human thyroid cancer cells, the gemcitabine incorporation within liposomes enhances the drug cytotoxic effect with respect to free gemcitabine, thus suggesting a more effective drug uptake inside the cells. This may allow the use of new formulations with lower dosages (side effect free) for the treatment of anaplastic human thyroid tumours.

## Background

At present, the prognosis of anaplastic thyroid carcinomas is very poor [1,2]. Since they are usually unable to concentrate radioiodine, the therapeutical approach is based on combination of aggressive surgery, external beam radiations and chemotherapy. Only rarely, however, this treatment results effective especially for treating metastatic disease [1,2]. A major limit of the chemotherapeutic agents presently used in these tumours, including doxorubicin, paclitaxel and various drug combinations [1,2], is represented by their low therapeutic index. Thus, the identification of systemic antineoplastic drugs effective against these carcinomas has particularly relevant implications.

Gemcitabine is a new fluorinated nucleoside analogue provided with a potent anti-tumour activity, tested in vitro and in vivo against a variety of solid malignancies [3-6]. In addition, gemcitabine is a potent radiosensitizing agent [7]. In a preclinical study, gemcitabine showed a marked cytotoxic activity against poorly differentiated human thyroid carcinoma cell lines [8]. In contrast, no appreciable response was observed in four patients with aggressive thyroid anaplastic carcinoma treated with combination of gemcitabine and vinorelbine [9]. Since hematological and other toxicities have been reported when effective anti-tumour doses of gemcitabine were tested [10], the aim of this study was to verify the possibility of maintaining the drug cytotoxicity at lower doses, by using a liposomal drug delivery system. Our data demonstrate that incorporation of gemcitabine in liposomes enhances the cytotoxic effect of the drug against a well-established human thyroid cancer cell line, as compared to the free drug.

## Methods

### Chemicals

Gemcitabine (2,2^1^-difluorodeoxycytidine) hydrochloride (HPLC purity >99%) was a kind gift of Eli-Lilly Italia S.p.A. (Sesto Fiorentino, Firenze, Italy) and was used without further purification. Cholesterol was obtained from Sigma Chemicals Co. (St. Louis, USA). 1,2-dipalmitoyl-sn-glycero-3-phosphocholine monohydrate and N-(carbonyl-methoxypolyethyleneglycol-2000)-1,2-distearoyl-sn-glycero-3-phosphoethanolamine sodium salt (MPEG-2000-DSPE) were purchased from Genzyme products (Suffolk, United Kingdom). Double-distilled pyrogen-free water from Sifra S.p.A. (Verona, Italy) was used. Sterile saline was a product of Frekenius Kabi Potenza S.r.l. (Verona, Italy). All other materials and solvents were of analytical grade (Carlo Erba, Milan, Italy).

### Liposome preparation

Liposome formulations were made up of DPPC/Chol/MPEG-2000-DSPE (8:3:1 molar ratio). To maximize the entrapment efficiency of a hydrophilic molecule such as gemcitabine, various liposome preparation procedures [11,12] were applied together. Namely, liposome colloidal suspensions were prepared by dissolving the lipid mixture (40 mg) in chloroform-methanol (3:1 v/v). Organic solvent was removed by a rotavapor thus allowing the formation of a thin lipid film on the inner surface of a pyrex glass vial. To remove any trace of organic solvent, lipid films were stored overnight in a Büchi T-50 under high vacuum at 30°C in the dark. Lipid films were hydrated with a solution (2 ml) of 250 mM ammonium sulphate by vortexing at 45°C for 15 min. The resulting colloidal suspension of multilamellar vesicles was submitted to ten cycles of freeze in liquid nitrogen and thaw in warm (35°C) water in order to allow the homogenous distribution of the ionic species. The entrapped ammonium sulphate solution was removed by centrifugation at 14000 rpm (IEC, International Equipment Company, mod MP4R, equipped with a mod. 851 rotor) for 1 h at a temperature of 4°C. The pellet was re-suspended in 400 μl of a 1 mM gemcitabine aqueous solution and stored at room temperature for 3 h. Double-distilled pyrogen-free water (1.6 ml) was added to the liposome suspension and then a freeze-drying procedure was carried out (Edwards freeze-dryer Modulayo). Then, the non-incorporated gemcitabine was removed and the freeze-dried liposomes were re-suspended just before the experiments with 2 ml of the culture medium. Scalar dilutions were prepared in the same medium to obtain the final concentrations used in the experiments.

### Liposome loading capacity

The loading capacity of liposomes was evaluated after removing the free gemcitabine (centrifugation at 14000 × g for 1 h at 4°C) from the vesicular colloidal suspension coming from the re-suspension of freeze-dried liposomes. The amount of gemcitabine in the supernatant was spectrophotometrically determined at 268.8 nm (Shimadzu UV-1601). UV calibration straight-line (*y *= 6.958 × 10^-3 ^+ 0.3971*x*, where *y *is the UV adsorbance and *x *is the drug concentration) presented a *r*^2 ^value of 0.9993. The amount of gemcitabine entrapped in liposomes was determined as a difference between the amount of drug added during liposome preparation and the amount of the entrapped drug present in the supernatant. The encapsulation efficiency was expressed as percentage of the total amount of gemcitabine that became entrapped.

### Cell cultures and cell viability

The ARO cells, from a human anaplastic thyroid carcinoma, were grown as previously described [13]. All experiments were performed in 12-well culture dishes, when cells reached approximately 50% confluence. Cell viability was evaluated by trypan bleu dye exclusion assay. Briefly, after incubation with gemcitabine or liposomes containing the drug at different doses, cells were trypsinized and the pellet re-suspended in a 0.4% trypan bleu buffer and counted in the hemocytometric chamber. Cell mortality was calculated as the percentage of stained cells over the total and expressed as the ratio between treated and untreated (control) cells. DNA cell content was assayed by using a fluorimetric DNA assay kit (Bio-rad laboratories, Segrate, Milano, Italy).

## Results

**Figure 1:**
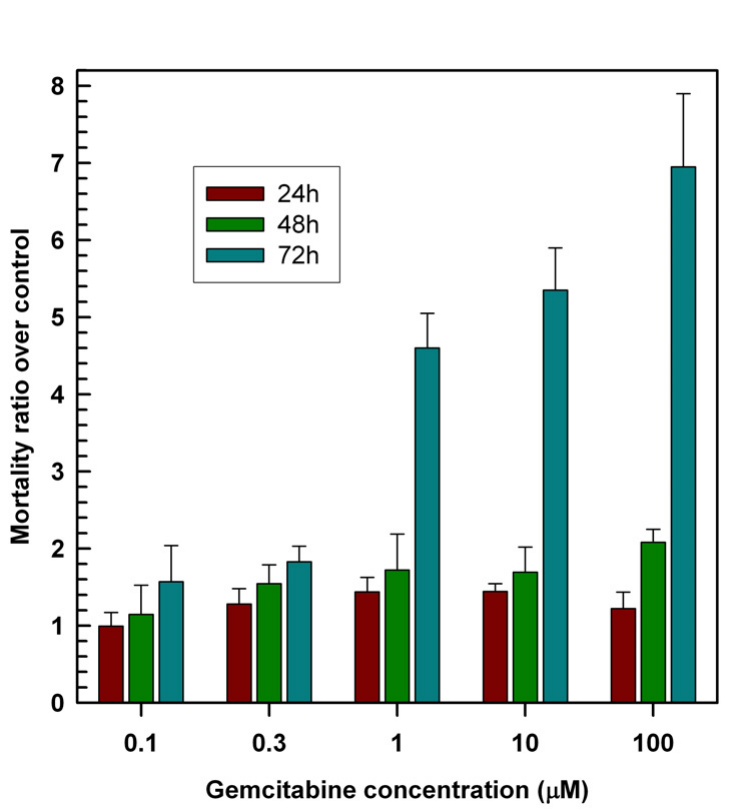
Cytotoxic effect of gemcitabine. ARO cells were grown to 50% confluence and treated with increasing concentrations of gemcitabine for different incubation time. Cell mortality was evaluated by trypan bleu dye exclusion assay (see Methods). In control cells, mortality was always lower than 5%. The values, expressed as the ratio over control, represent the mean ± SEM of at least three different experiments in triplicates.

We first analysed the effects of different concentrations of gemcitabine on the viability of ARO thyroid cancer cells. As shown in Figure 1 the cytotoxic effect was observed only after 48 or 72 h incubation of the drug. After 72 h, the cell mortality increased 4.6 and 7.9 fold over control using 1 and 100 μM of gemcitabine. In accordance with previous reports [8], no significant effect was visible after 24 h exposure (Fig. 1).

**Figure 2:**
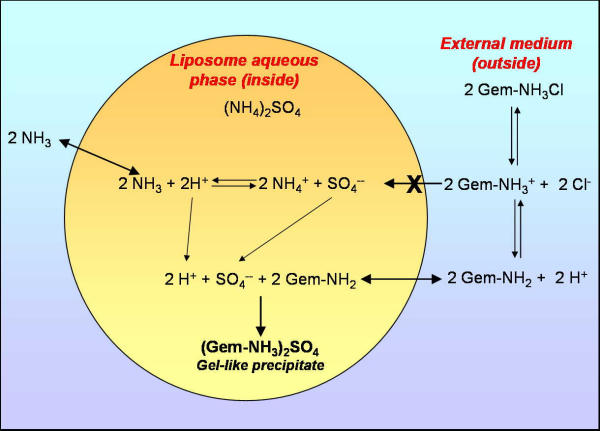
Schematic representation of the gemcitabine encapsulation process within liposomes by means of the presence of a pH gradient elicited by the co-encapsulation of a 250 mM ammonium sulphate solution.

In order to improve the drug entry into the cells, gemcitabine entrapment in a liposome capsule was next performed, using a combination of various liposome preparation procedures. In particular, a pH gradient method was used [14]. Namely, the presence of ammonium sulphate in the internal compartments of liposomes provide an acidic environment that elicit the protonation of gemcitabine in order to drastically reduce the drug back-diffusion (leakage) from liposomes (Figure 2). The combination of various liposome preparation procedures together with the application of a pH gradient provided an encapsulation efficiency of ~90%.

**Figure 3:**
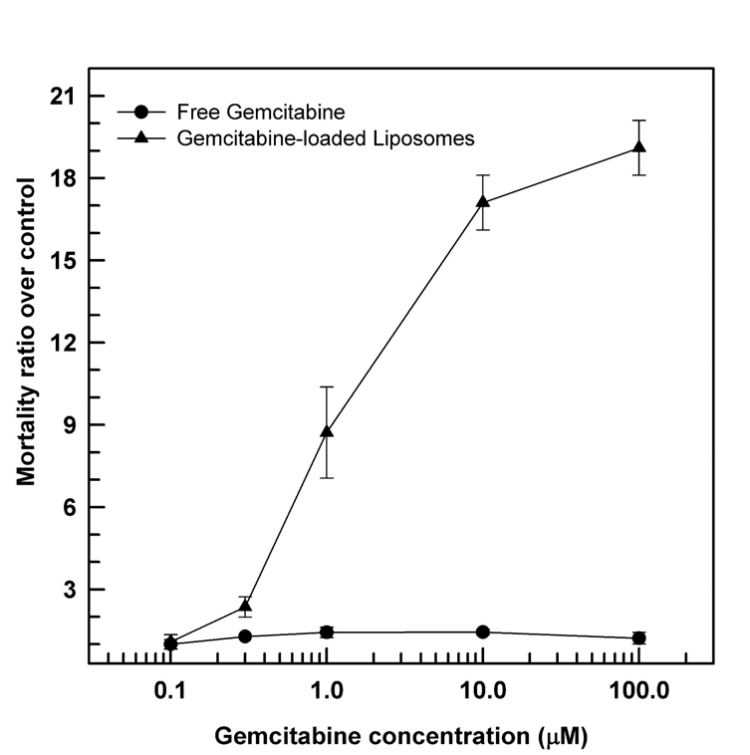
Cytotoxic effect of gemcitabine-loaded liposomes. ARO cells were grown to 50% confluence and treated with increasing concentrations of either free gemcitabine or gemcitabine-loaded liposomes for 24 h. Cell mortality was evaluated by trypan bleu dye exclusion assay (see Methods). The values, expressed as the ratio over control, represent the mean ± SEM of at least three different experiments in triplicates.

The gemcitabine-loaded liposomes were then tested for their cytotoxic activity against ARO cell, and the results compared with those of gemcitabine alone. As shown in Figure 3, a cytotoxic effect appeared after 24 h incubation, with an initial increased cell mortality at 0.3 μM (2.4 fold over control), and a maximum at 10 μM (death of almost all the cells) (Figure 3). Similar results were obtained by analysing the DNA content of ARO cells treated with free or liposome-encapsulated gemcitabine (data not shown).

## Discussion

Despite an intense application of aggressive multimodal therapeutic regimens, no standardized protocol has shown success in the treatment of anaplastic thyroid carcinoma [2]. A critical need for new approaches is therefore vital for the systemic therapy of metastatic anaplastic thyroid carcinoma. At present one promising option is provided by the gene therapy. Studies are currently in progress attempting to: a. reintroducing iodine uptake by viral-driven Iodide/sodium symporter gene expression [15,16]; b, inducing tumour cell death by expressing p53 or by suicide gene prodrug systems [17,18]. An alternative approach is represented by increasing the effectiveness of chemotherapy, using novel agents, combination therapy or enhancing drug delivery inside the tumour cell.

The latter option has been taken advantages in the progress related to liposome research. Traditional dosage forms of anticancer drugs have the limitation of many potential obstacles (barriers) before they reach their target site, one of these is the large volume of distribution after i.v. administration. This situation elicits a low therapeutic index and a high toxicity in healthy tissues [19]. After encapsulation into liposomes similar to those used in our investigation the volume of distribution of a drug is reduced, and its concentration in the tumour tissue is increased [20]. In fact, liposomes can protect drugs from metabolic inactivation, and, due to size limitation, they are not able to transport encapsulated molecules across healthy endothelium, thus avoiding anticancer drug accumulation in a large extent in healthy tissues and hence a decrease of side-effects [21]. On the other hand, the increased permeability of the tumour endothelium allows the extravasation of liposomes, with a consequent accumulation of anticancer drugs in the site of action [22]. Furthermore, it should be considered that liposomes are non-toxic being mostly constituted by naturally occurring lipids.

In the last years, gemcitabine has emerged as a very potent anti-tumour drug and it is currently used, alone or in combination, in the treatment of patients with different malignancies, including ovarian, pancreatic, non-small cell lung and other cancers [3-6]. Despite its promising effectiveness derived from a preclinical study [8], no appreciable response was observed in a clinical study including four patients with aggressive thyroid anaplastic carcinoma treated with combination of gemcitabine and vinorelbine [9]. Although low, compared to other anti-neoplastic drugs, hematological and other toxicities appeared when effective anti-tumour doses of gemcitabine were tested.

In this study, we prepared a liposome formulation with a high entrapment efficiency of gemcitabine and tested its efficacy against a well established human poorly differentiated thyroid carcinoma cell line. Our data demonstrate that, in ARO cells, incorporation of gemcitabine in liposomes enhances the cytotoxic effect of the drug as compared to free gemcitabine, suggesting a more effective uptake of the drug inside the cells. This activity was demonstrated at concentrations even lower than serum levels obtained in clinical trials. In our knowledge, no data are available in the literature concerning the relationship between gemcitabine uptake and its cytotoxicity, since neither liposomes or other carrier system have been tested against tumour cells. Our preliminary experiments (unpublished observations) show that a similar effect (improved uptake and cytotoxicity at lower doses) may be obtained also in colon cancer cells. It is note worthing that the various component used for the preparation of the liposomal delivery device were already approved by regulatory offices to be used in the preparation of liposome-based formulations, i.e. Doxil^®^.

## Conclusion

The clinical predictive value of the in vitro cell line preclinical cancer model is well established [23] and the ARO cells are one of the cell model of anaplastic thyroid cancer more exploited both for investigating the mechanism of tumour progression and for testing new molecules with antiproliferative effect. However, these promising observations need to be confirmed in studies using in vivo xenograft cancer models, prior to propose the use of such preparations at lower (presumably side effect free) doses in a clinical trial of human thyroid tumours.

## Competing interests (medicine)

None declared.

## Pre-publication history

The pre-publication history for this paper can be accessed here:
